# Clinical relevance of depressed kynurenine pathway in episodic migraine patients: potential prognostic markers in the peripheral plasma during the interictal period

**DOI:** 10.1186/s10194-021-01239-1

**Published:** 2021-06-25

**Authors:** Bernadett Tuka, Aliz Nyári, Edina Katalin Cseh, Tamás Körtési, Dániel Veréb, Ferenc Tömösi, Gábor Kecskeméti, Tamás Janáky, János Tajti, László Vécsei

**Affiliations:** 1 Department of Neurology, Faculty of Medicine, University of Szeged, Semmelweis u 6, Szeged, H6725 Hungary; 2 MTA-SZTE Neuroscience Research Group, Department of Neurology, Faculty of Medicine, University of Szeged, Szeged, Hungary; 3 Faculty of Health Sciences and Social Studies, University of Szeged, Szeged, Hungary; 4 Department of Radiology, Faculty of Medicine, University of Szeged, Szeged, Hungary; 5 Department of Medical Chemistry, Interdisciplinary Excellence Centre, University of Szeged, Szeged, Hungary; 6 Department of Neurology, Interdisciplinary Excellence Centre, University of Szeged, Szeged, Hungary

**Keywords:** Episodic migraine, Interictal-ictal periods, Plasma tryptophan metabolites: kynurenine-serotonin-melatonin pathways, Aura-without aura, Clinical features

## Abstract

**Background:**

Altered glutamatergic neurotransmission and neuropeptide levels play a central role in migraine pathomechanism. Previously, we confirmed that kynurenic acid, an endogenous glutamatergic antagonist, was able to decrease the expression of pituitary adenylate cyclase-activating polypeptide 1–38, a neuropeptide with known migraine-inducing properties. Hence, our aim was to reveal the role of the peripheral kynurenine pathway (KP) in episodic migraineurs. We focused on the complete tryptophan (Trp) catabolism, which comprises the serotonin and melatonin routes in addition to kynurenine metabolites. We investigated the relationship between metabolic alterations and clinical characteristics of migraine patients.

**Methods:**

Female migraine patients aged between 25 and 50 years (*n* = 50) and healthy control subjects (*n* = 34) participated in this study. Blood samples were collected from the cubital veins of subjects (during both the interictal/ictal periods in migraineurs, *n* = 47/12, respectively). 12 metabolites of Trp pathway were determined by neurochemical measurements (UHPLC-MS/MS).

**Results:**

Plasma concentrations of the most Trp metabolites were remarkably decreased in the interictal period of migraineurs compared to healthy control subjects, especially in the migraine without aura (MWoA) subgroup: Trp (*p* < 0.025), L-kynurenine (*p* < 0.001), kynurenic acid (*p* < 0.016), anthranilic acid (*p* < 0.007), picolinic acid (*p* < 0.03), 5-hydroxy-indoleaceticacid (p < 0.025) and melatonin (*p* < 0.023). Several metabolites showed a tendency to elevate during the ictal phase, but this was significant only in the cases of anthranilic acid, 5-hydroxy-indoleaceticacid and melatonin in MWoA patients. In the same subgroup, higher interictal kynurenic acid levels were identified in patients whose headache was severe and not related to their menstruation cycle. Negative linear correlation was detected between the interictal levels of xanthurenic acid/melatonin and attack frequency. Positive associations were found between the ictal 3-hydroxykynurenine levels and the beginning of attacks, just as between ictal picolinic acid levels and last attack before ictal sampling.

**Conclusions:**

Our results suggest that there is a widespread metabolic imbalance in migraineurs, which manifests in a completely depressed peripheral Trp catabolism during the interictal period. It might act as trigger for the migraine attack, contributing to glutamate excess induced neurotoxicity and generalised hyperexcitability. This data can draw attention to the clinical relevance of KP in migraine.

**Supplementary Information:**

The online version contains supplementary material available at 10.1186/s10194-021-01239-1.

## Introduction

Migraine is one of the most frequent primary headache disorder associated with high socioeconomic impact. The pathomechanism is complex and several factors control the development of attacks, among others disturbances of glutamatergic neurotransmission can lead to excitotoxicity and generalised neuronal hyperexcitability, which underlie the formation of migraine [1–3]. In this study, we focused on the investigation of kynurenine pathway (KP), which is closely related to the glutamatergic system [4], and potentially interacts with neuropeptides as well [5]. Therefore, kynurenine metabolites may be relevant as biomarkers and/or therapeutic targets for migraine.

KP is the main metabolic route (95%) of tryptophan (Trp) in parallel with the synthesis of serotonin (5-hydroxytryptamine, 5-HT) and melatonin (N-acetyl-5-methoxytrypamine, MELA). Since all three molecules are involved in migraine-related processes [6–11], the question arises whether metabolites of kynurenine contribute to or are affected by these processes. The KP unfolds as follows: firstly, L-kynurenine (KYN) is formed from Trp indirectly during an oxidative step, then three different enzymes determine the generation of further metabolites: 1) kynurenine aminotransferases (KATs) are able to form kynurenic acid (KYNA) from KYN, 2) the kynurenine monooxygenase can generate 3-hydroxykynurenine (3-HK) followed by the formation of xanthurenic acid (XA) and 3-hydroxy-anthranilic acid (3-HANA), and 3) the kynureninase is able to form anthranilic acid (ANA), which can transform to 3-HANA again that generates quinolinic acid (QUINA) and picolinic acid (PICA) in further indirect steps. Several metabolites are neuroactive, but they have different sources and actions: e.g. KYNA is synthetised in astrocytes and behaves as a neuroprotective agent, while QUINA is the product of activated microglial cells/macrophages and it is considered a neurotoxic compound for the central nervous system (CNS) [12] (Fig. 6).

These metabolites are present in the nano/micromolar range in the human brain and they act partly on the glutamatergic system [13, 14]. Both ionotropic and metabotropic glutamate receptors are involved in mechanisms related to migraine [15, 16], but N-methyl-D-aspartate (NMDA) receptors have a pronounced role in the onset of cortical spreading depression (CSD) [17, 18], the development of hyperalgesia [19] and the activation of migraine generators [20, 21]. Since the inhibition of NMDA receptors is believed to protect against glutamate-caused excitotoxicity and KYNA has a competitive antagonist effect on these receptors [4], it is possible that the KP has therapeutic potential in headache disorders. Moreover, KYNA exhibits affinity to other receptors as well (e.g. α-amino-3-hydroxy-5-methyl-4-isoxazole propionic acid receptors, G protein coupled receptor 35, aryl hydrocarbon receptors, α7-nicotinic acetylcholine receptor), enabling widespread modulating properties [22]. Downregulated KP was observed in different animal models of TS activation [23–26], while administration of KYN, KYNA and certain KYNA-derivatives seemed effective in preventing migraine-related neuronal, molecular and electrophysiological alterations in preclinical studies [5, 27–30].

However, human studies about the function of the KP in headache diseases are scarce: Curto and co-workers were the first who measured the metabolites of the KP in the serum of patients diagnosed with chronic migraine and cluster headache [31, 32]. They demonstrated that chronic migraine is associated with abnormalities of the KP compared to healthy controls and emphasised the significance of ANA. However, a systematic and detailed examination, which contains the 1) determination of not only the KP, but also main peripheral Trp metabolites with particular regard to the influence of 2) interictal/ictal periods and 3) clinical features of episodic migraine patients is needed which can help us understand the role of kynurenine metabolites and develop new, potential strategies in the diagnosis and therapy of migraine.

Our aims were:

- to determine the concentrations of main metabolites of Trp pathway (5-HT, MELA and KP) in the peripheral plasma of episodic migraine patients compared to healthy control subjects.
- to distinguish between metabolic alterations in the interictal/ictal periods and in the two subgroups of patients (migraine with and without aura)
- to describe the relationship between altered Trp metabolism and clinical features of the disease/attacks.

## Materials and methods

### Participants

All patients enrolled in this study are treated as outpatients at the Department of Neurology, Faculty of Medicine and University of Szeged. Investigations were conducted after the approval of the local Ethical Committee of the University of Szeged (87/2009) and the Department of Health Administration of National Public Health Centre (29022–5/2019/EÜIG, 28324–5/2019/EÜIG) adhering to the most recent revision of the Declaration of Helsinki.

Inclusion criteria: Episodic migraine patients (EM, *n* = 50) fulfilling the criteria of the 3rd edition of The International Classification of Headache Disorders were registered, and healthy control subjects (*n* = 34) were recruited. In order to keep the groups as homogenous as possible, they were matched in terms of age and sex (25–50 years, female).

Peripheral blood samples were collected from the cubital vein of patients during the interictal (attack free) and ictal (attack) periods, and from healthy controls on one occasion. Ethylenediaminetetraacetic acide containing blood collection tubes (BD Vacutainer K2E 6 ml) were used to obtain the samples between 8:00 a.m. and 2:00 p.m. (except the ictal samples, which were acquired during attacks between 7:00 a.m. and 7:00 p.m.). Plasma samples were separated (3000 rpm at 4 °C for 15 min) and stored at − 80 °C until determination of Trp metabolites by ultra high-performance liquid chromatography–tandem mass spectrometry (UHPLC–MS/MS). Samples were coded to allow for blind measurements.

From among the 50 migraineurs, 47 samples were acquired during the interictal phase and 12 samples were acquired during the ictal phase (all samples derived from different patients). Self-controlled paired samples were taken from 10 migraine patients during both periods. Interictal phase was defined as headache-free period at least 48 h after the last attack, considering the possibility of postdrome phase. During headache attacks, patients were asked not to take their usual painkillers or specific migraine attack medication until blood samples were taken. There were no restrictions regarding food and drink intake. Exclusion criteria for both the EM and control group included the presence of other type of headache (e.g. tension type headache) less than 48 h before sampling or other chronic pain conditions related to traumatic events or serious systemic disorders such as cardiovascular and metabolic diseases, immunological, neurological disorders, as well as clinically diagnosed psychiatric disorders. Subsequently, using any kind of chronic medication was also not allowed. We used the Hamilton’s depression scale before samplings to screen participants for depression. Detailed headache questionnaires were taken from migraineurs regarding the duration of disease and attacks, the frequency of attacks, the subjective feeling of the intensity of pain measured by a numeric Visual Analogue Scale (VAS), the presence of allodynia, provocative factors, prodromal- and accompanying symptoms, the relationship between hormonal changes (menstruation cycle, contraceptive drugs) and migraine attacks and the occurrence of migraine in the family. Table 1 contains relevant demographic and clinical data.

**Table 1:** General and clinical data of migraineurs and control subjects (median ± interquartile range)

	Episodic migraine patients	Healthy control subjects
**Gender**	Female	Female
**Age (years)**	33.22 ± 9.07	30.50 ± 12.77
**Number of patients/samples in different subgroups according to the different comparisons**	**All patients, n = 50** Interictal sample, n = 38 Ictal sample, n = 12	n = 34 No regular headache syndromes, chronic diseases and drugs.
	**All interictal patients, n = 47** Migraine with aura samples, n = 11 Migraine without aura samples, n = 36	
	**All Migraine without aura patients n = 38** Interictal samples, n = 28 Ictal samples, n = 10	
**Clinical data of Migraine without aura patients** **(n**_**interictal samples**_ **= 36, n**_**ictal samples**_ **= 10)**		
**Disease Duration (years)**		12.50 ± 15.00
**Attack frequency (attack/year)**		29.50 ± 29.50
**Visual analogue scale (VAS)**		moderate n = 7, severe n = 29
**Allodynia**		No n = 22, Yes n = 14
**Menstruation**		No n = 20, Yes n = 16
**Contraceptives**		No n = 25, Yes n = 11
**Prophylactic therapy before the interictal sampling**		No n = 27, Yes n = 9 (iprazochrome + magnesium orotate n = 4, topiramate n = 4, valproic acid n = 1)
**Regular attack therapy**		eletriptan, sumatriptan > diclophenac > acetylsalicylic acid + metoclopramide, indometacin, nimesulid, naproxen
**Last attack before the interictal cupping (days)**		7.00 ± 9.00
**Prophylactic therapy before ictal cupping**		No n = 7, Yes n = 3 (iprazochrome + magnesium orotate n = 1, topiramate n = 2
**Ictal sampling (ICHD3**^**rd**^ **symptoms)**	**Beginning of attack (hours)** **Severity of pain (VAS)** **Previous last attack (days)**	7.50 ± 18.75
		8.00 ± 1.00
		16.50 ± 14.00

### Quantitative determination of Trp metabolites by UHPLC–MS/MS – method validation

Recently, we have published a paper [33] on the development of a new, robust UHPLC–MS/MS method for simultaneous quantitative characterization of Trp and 11 of its metabolites. The method was applied for quantification of these metabolites in cerebrospinal fluid and serum samples of patients with multiple sclerosis and now for plasma samples of migraineurs. Briefly, plasma samples were deproteinized with acetone–methanol mixture containing stable isotope labeled form of 12 metabolites as internal standards. Half of the supernatants were esterified with n-butanol and then remixed with the untreated fraction to analyze butylated 3-HK, PICA, and QUIN in the same chromatographic run with nine underivatized metabolites. Chromatographic separation was performed on a pentafluorophenyl column (Phenomenex, Torrance, CA, US) using formic acid containing water–methanol gradient solvent system. All mass spectrometric measurements were conducted using the Q Exactive™ Plus Hybrid Quadrupole-Orbitrap Mass Spectrometer (Thermo Fisher Scientific, San Jose, CA, USA) connected online to an ACQUITY I-Class UPLC™ liquid chromatography system (Waters, Manchester, UK). As in our recent publication [33], the validation process was carried out on serum and cerebrospinal fluid samples, we decided to validate the method for plasma samples too. For the validation process, the linearity, limits of detection (LOD) and quantification (LOQ), precision, accuracy, and recovery were assessed following the official guidelines.

### Statistical analysis

We performed several different analyses to make sure all clinical parameters were considered. Firstly, all patients (migraine with aura (MWA) or without aura (MWoA)) were analysed together, forming three groups: healthy control, interictal and ictal phase patients. In all cases, the normality and homogeneity of variance were tested; afterwards, Kruskal-Wallis tests were run. Additionally, we assessed group differences using two robust probes, which test the equality of group variances (Welch- and Brown-Forsythe tests). Thereafter, we divided migraineurs in two subgroups according to the presence of aura, and compared the interictal data of these subgroups to healthy subjects separately. For later analyses, we only used the MWoA group data due to the sample size and the relevant differences identified in previous statistical tests. The effect of clinical parameters (disease duration, attack frequency, VAS, presence of allodynia, involvement of menstruation cycle, applied therapies and age) on the metabolic changes was investigated only in the MWoA subgroup using linear regression models and two independent samples analyses. Median of data and value of interquartile range (IQR) were represented in the figures. Significance level was accepted at *p* < 0.05.

Furthermore, we opted to confirm our results in a multivariate setting, since the metabolites in this study are parts of interconnected pathways and exhibit high interdependence and cross-correlation. Specifically, we asked whether participants could be accurately classified as migraine patient or healthy participant in our sample based on all measured interictal metabolite levels, and which metabolites are (most) important in making this distinction. PLS models are suitable in datasets where variables exhibit multicollinearity [34], and can be extended to a LDA model by using the latent variables estimated during the PLS analysis, a classification method widely used in metabolomics and chemometrics [35]. As the details of the PLS-LDA method are described elsewhere [36], we provide a brief delineation in Supplementary Description 1. Only participants with a complete metabolite profile were included in the PLS-LDA model (n_healthy_ = 30, n_migraine_ = 37). Age was also included as an independent variable to account for the possible effect it has on metabolite levels. The PLS-LDA analysis was performed in MATLAB R2012 (MathWorks, Inc.) using the libPLS MATLAB package (libPLS version 1.98 [36], www.libpls.net).

## Results

### Method validation

The LOD, LOQ, retention time, and linearity of the calibration curves with r^2^ values are shown in Suppl. Table 1 for plasma. For all analytes, r^2^ exceeded 0.99. In each case, LODs were less than 9.75 nM. The intra- and inter-day precision values of the method for plasma (Suppl. Table 2 and Table 3) lead to the conclusion that the developed method has reliable precision. The accuracy ranged from 85.1 to 114.8% (Suppl. Table 2 and Table 3), whereas the recoveries of the analytics ranged between 90.28 and 101.81% for “blank” plasma (Suppl. Table 4). Our values are in the range recommended by the official guidelines [ICH, FDA]. Concentrations of 12 metabolites (Trp, KYN, KYNA, ANA, 3-HK, XA, 3-HANA, PICA, QUINA, 5-HT, 5-HIAA, MELA) of the KP starting from Trp were determined during this experiment. Levels of 5-HT and MELA were undetectable in some samples; therefore, their sample sizes for measurements are described separately.

### Differences in plasma levels of Trp metabolites between interictal/ictal periods of migraineurs and healthy controls

**Fig. 1:**
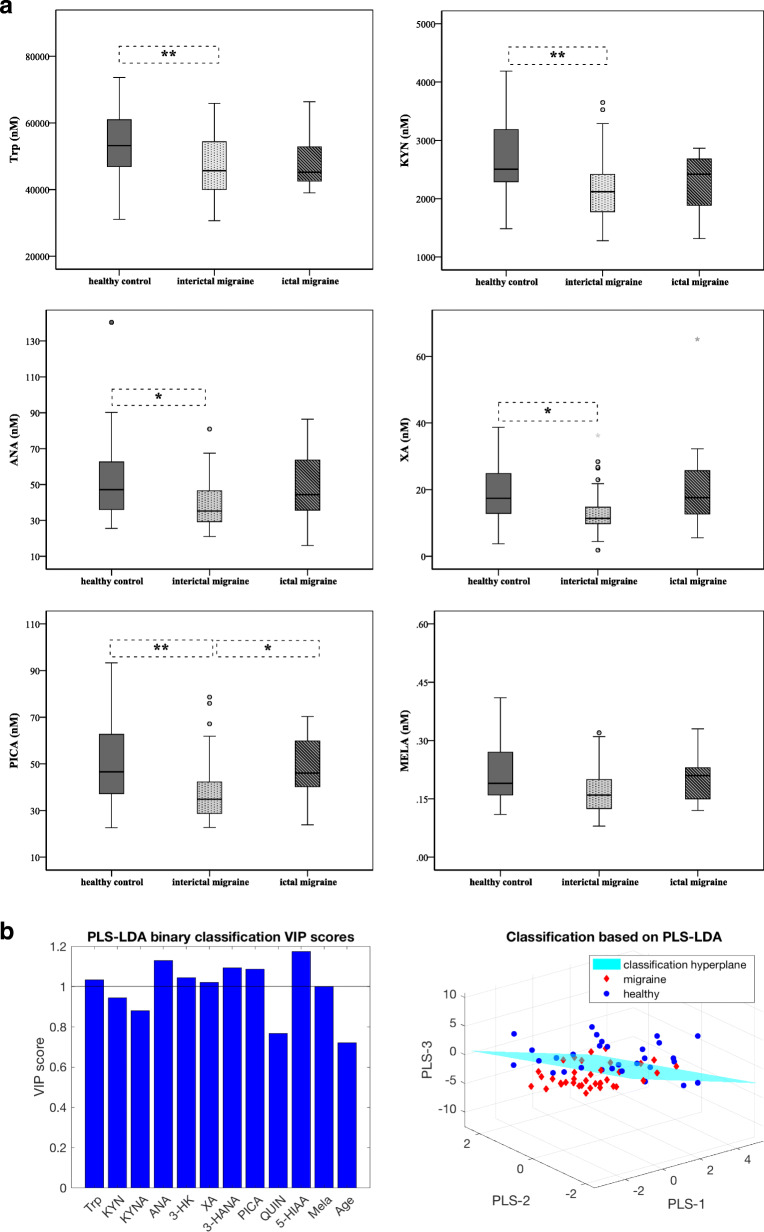
**a.** Plasma concentrations of Trp, KYN, ANA, XA, PICA and MELA in controls and migraineurs in both interictal/ictal periods (median, interquartile range, minimum, maximum values and outliers were presented in this figure). Sample sizes in groups: healthy control (*n* = 34), interictal phase of migraineurs (*n* = 38), ictal phase of migraineurs (*n* = 12). Sample sizes in case of MELA: healthy control (*n* = 30), interictal phase of migraineurs (*n* = 37), ictal phase of migraineurs (*n* = 10). Significance levels: * *p* < 0.05, ** *p* < 0.01. Trp: Tryptophan, KYN: L-kynurenine, ANA: Anthranilic acid, XA: Xanthurenic acid, PICA: Picolinic acid, MELA: Melatonin. **b.** Results of the partial least squares – linear discriminant analysis (PLS-LDA). Left: The bar plot depicts variable importance projection (VIP) scores of variables used in the PLS decomposition. Right: 3D-projection plots of migraine and healthy metabolite profiles in the PLS latent variable space. The classification hyperplane was obtained using linear discriminant analysis. Trp: Tryptophan, KYN: L-kynurenine, KYNA: Kynurenic acid, ANA: Anthranilic acid, 3-HK: 3-hydroxykynurenine, XA: Xanthurenic acid, 3-HANA: 3-hydroxy-anthranilic acid, PICA: Picolinic acid, QUINA: Quinolinic acid, 5-HIAA: 5-hydroxy-indoleaceticacid, MELA: Melatonin, PLS-LDA: partial least squares–linear discriminant analysis

We detected significantly lower plasma concentrations of Trp metabolites (Trp, KYN, ANA, XA and PICA) in the interictal phase of migraineurs (*n* = 38) compared to the healthy control group (*n* = 34). MELA showed a similar tendency but did not reach statistical significance (n_control_ = 30 vs. n_interictal_ = 37 vs. n_ictal_ = 10). Details are included in Table 2. A tendency of elevated Trp metabolite levels were revealed in the ictal phase of migraineurs (*n* = 12) compared to the attack free period but only PICA levels differed significantly (34.86 ± 13.73 vs. 46.04 ± 24.39; *p* < 0.049) (Fig. 1a). Level of 5-HT showed opposite alterations between controls (*n* = 17) and patients (n_interictal_ = 25, n_ictal_ = 8) compared to other metabolites, but the difference was not significant in any of the cases. The interictal data of 10 migraineurs, whose plasma samples were collected from both periods, were excluded from this analysis to avoid statistical problems.

**Table 2:** Plasma concentrations of Trp metabolites in healthy controls and migraineurs in both interictal and ictal periods

Metab. (nM)	Groups: Median ± IQR			***p***-values between groups
	0. Healthy Control	1. Interictal Migraine	2. Ictal Migraine	
**Trp**	53,203.65 ± 14,599.34	45,657.54 ± 4711.21	45,210.69 ± 11,292.49	0–1: **0.008**
**KYN**	2505.77 ± 902.53	2119.41 ± 648.54	2418.99 ± 819.22	0–1: **0.003**
**KYNA**	39.59 ± 15.32	33.56 ± 15.64	36.01 ± 10.57	0–1: 0.061
**ANA**	47.12 ± 27.79	35.23 ± 18.88	44.39 ± 28.61	0–1: **0.017**
**3-HK**	47.29 ± 27.89	44.79 ± 18.73	48.41 ± 34.54	0–1: 0.511
**XA**	17.42 ± 13.46	11.39 ± 5.31	17.61 ± 14.66	0–1: **0.02**
**3-HANA**	47.78 ± 24.59	44.85 ± 23.61	42.40 ± 21.24	0–1: 0.733
**PICA**	46.61 ± 25.63	34.86 ± 13.73	46.04 ± 24.39	0–1: **0.005** 1–2: **0.049**
**QUINA**	252.92 ± 169.69	190.86 ± 97.12	239.66 ± 178.05	0–1: 0.096
**5-HT**	468.11 ± 710.24	772.24 ± 493.89	491.23 ± 509.87	0–1: 0.243
**5-HIAA**	45.31 ± 20.46	37.46 ± 11.19	42.67 ± 11.10	0–1: 0.063
**MELA**	0.19 ± 0.11	0.16 ± 0.09	0.21 ± 0.10	0–1: 0.053

All *p*-values were added in the table between control and interictal group of migraine patients. Significant difference between phases of migraine was revealed only in case of PICA

*IQR: interquartile range, Metab.: metabolites, Trp: Tryptophan, KYN: L-kynurenine, KYNA: Kynurenic acid, ANA: Anthranilic acid, 3-HK: 3-hydroxikynurenine, XA: Xanthurenic acid, 3-HANA: 3-hydroxi-anthranilic acid, PICA: Picolinic acid, QUINA: Quinolinic acid, 5-HT: 5-hydroxytryptamine, 5-HIAA: 5-hydroxi-indoleaceticacid, MELA: Melatonin*

The PLS-LDA model achieved the best classification results with 3 latent variables extracted in the PLS decomposition, with a sensitivity of 63.33%, a specificity of 75.68% and an AUC of 0.84 (RMSE = 0.29). Metabolites which proved most definitive in the classification (chosen as having a VIP of > 1; see the Supplementary Description 1 for further details) were similar to those that showed alterations in the migraine group, namely Trp, ANA, 3-HK, XA, 3-HANA, PICA, 5-HIAA, MELA (Fig. 1b).

### Differences in plasma levels of Trp metabolites between migraine with aura/without aura and healthy controls

**Fig. 2:**
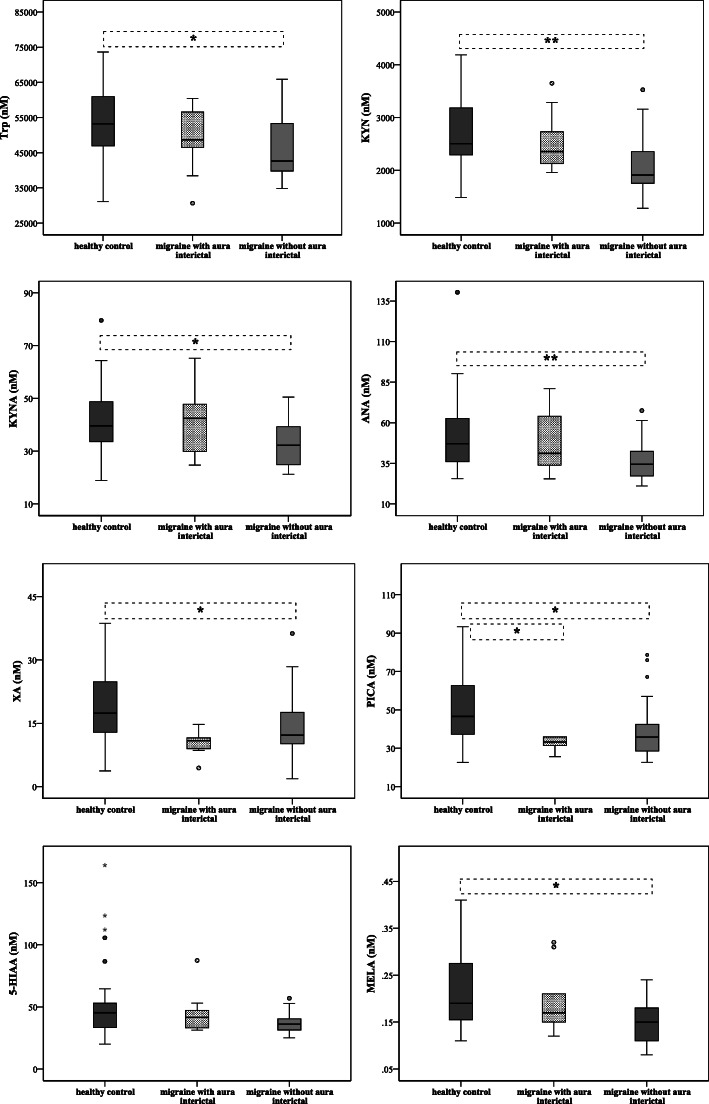
Differences in plasma levels of Trp, KYN, KYNA, ANA, XA, PICA, 5-HIAA and MELA between patients with aura (MWA) and without aura (MWoA) (median, interquartile range, minimum, maximum values and outliers were presented in this figure). Sample sizes in groups: healthy control (n = 34), interictal phase of MWA (*n* = 11), interictal phase of MWoA (*n* = 36). Sample sizes in case of MELA: healthy control (n = 30), interictal phase of MWA (*n* = 9), interictal phase of MWoA (*n* = 28). Significance levels: * p < 0.05, ** p < 0.01. Trp: Tryptophan, KYN: L-kynurenine, KYNA: Kynurenic acid, ANA: Anthranilic acid, XA: Xanthurenic acid, PICA: Picolinic acid, 5-HIAA: 5-hydroxy-indoleaceticacid, MELA: Melatonin

To see whether Trp metabolism differs in the presence of aura, we divided the patients into two subgroups: MWA (*n* = 11) and MWoA (*n* = 36). Decreased interictal plasma concentrations of Trp metabolites were measured in both subgroups of migraineurs (except of 5-HT), but significant alterations were detected in the Trp, KYN, KYNA, ANA, XA, PICA and MELA levels in the MWoA group compared to healthy controls (*n* = 34). In MWA patients, only the expression of PICA was significantly lower compared to controls (33.72 ± 17.84 vs. 46.61 ± 25.63; *p* < 0.038). Details can be seen in the Fig. 2 and Suppl. Table 5.

### Differences in plasma levels of Trp metabolites between groups of interictal/ictal periods of migraine without aura and healthy controls

**Fig. 3:**
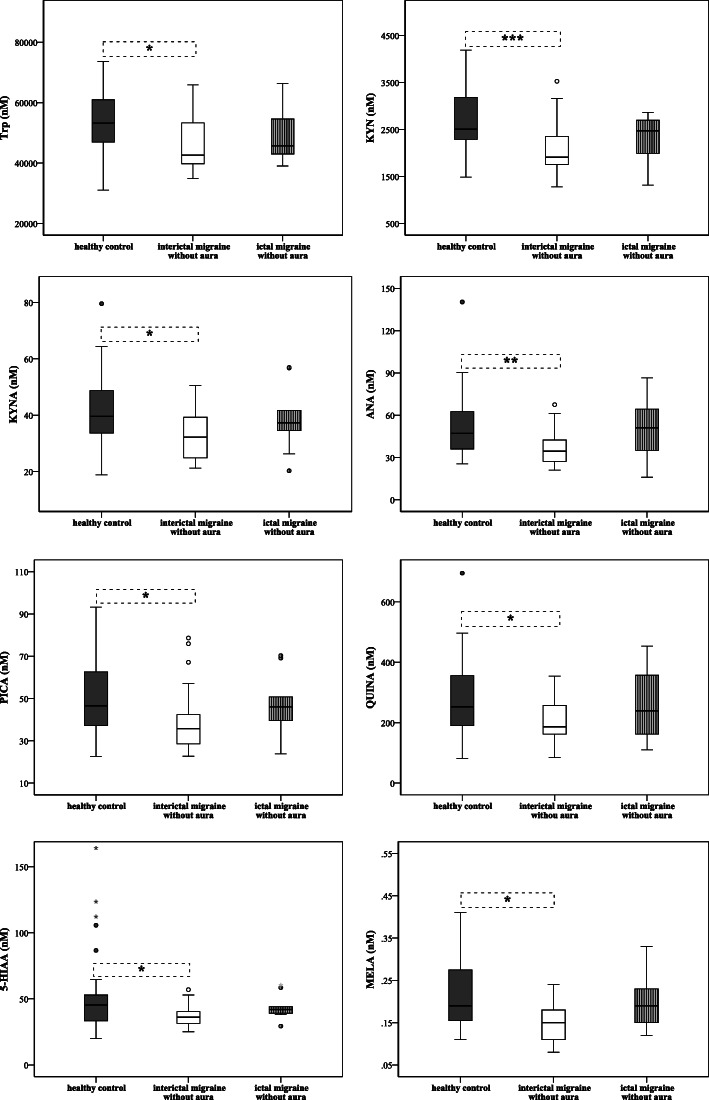
Differences in plasma levels of Trp, KYN, KYNA, ANA, PICA, QUINA, 5-HIAA and MELA between groups of interictal/ictal phases of migraine without aura patients and healthy subjects (median, interquartile range, minimum, maximum values and outliers were presented in this figure). Sample sizes in groups: healthy control (n = 34), interictal phase of MWoA (n = 28), ictal phase of MWoA (n = 10). Sample sizes in case of MELA: healthy control (n = 30), interictal phase of MWoA (*n* = 22), ictal phase of MWoA (n = 9). Significance levels: * p < 0.05, ** p < 0.01, *** *p* < 0.001. Trp: Tryptophan, KYN: L-kynurenine, KYNA: Kynurenic acid, ANA: Anthranilic acid, PICA: Picolinic acid, QUINA: Quinolinic acid, 5-HIAA: 5-hydroxy-indoleaceticacid, MELA: Melatonin

According to the results of previous comparisons, only the data of MWoA patients were analysed further. Significantly lower plasma concentrations of Trp metabolites (Trp_80%_, KYN_76%_, KYNA_81%_, ANA_73%_, PICA_77%_, QUINA_74%_, 5-HIAA_80%_ and MELA_79%_) were detected in the interictal phase of patients (*n* = 28) compared to healthy subjects (*n* = 34, c_metabolites_ = 100%). During the ictal period (*n* = 10) the concentrations of Trp_86%_, KYN_99%_, KYNA_94%_, ANA_109%_, PICA_99%_, QUINA_95%_, 5-HIAA_93%_ and MELA_100%_ tended to show higher levels or in some cases (ANA_109%_, 3-HK_116%_ and XA_101%_) the concentrations were over the control (Fig. 3 and Suppl. Table 6). Two independent samples comparison showed that concentrations of ANA (34.45 ± 15.95 vs. 51.18 ± 33.64, *p* < 0.040), 5-HIAA (36.14 ± 9.26 vs. 41.95 ± 8.83, *p* < 0.030) and MELA (0.15 ± 0.07 vs. 0.19 ± 0.08, *p* < 0.37) increased significantly during the ictal period compared to the attack free phase in MWoA patients.

### Associations between altered plasma Trp metabolites in the interictal periods of migraine without aura patients and clinical parameters

**Fig. 4:**
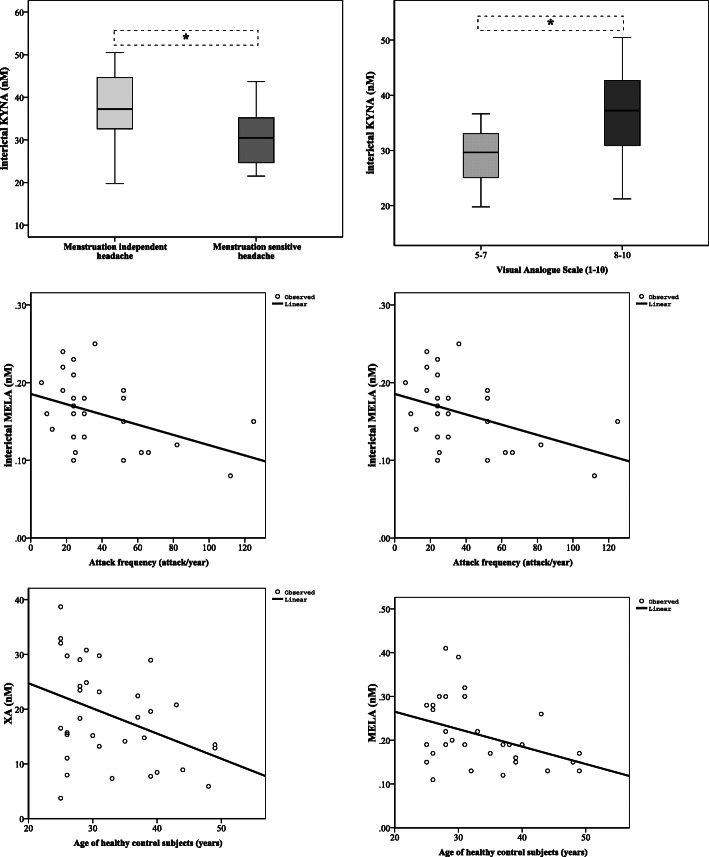
Associations between metabolic changes and clinical features of MWoA patients in the interictal period, and age of healthy controls (median, interquartile range, minimum and maximum values were presented in the figures of KYNA). Sample sizes in groups: interictal phase of migraineurs without aura (n = 36); Sample size in case of MELA: healthy control (n = 30), interictal phase of migraineurs without aura (n = 28). KYNA: Kynurenic acid, XA: Xanthurenic acid, MELA: Melatonin

Altered KYNA concentrations were identified in different subgroups of 36 interictal migraineurs according to the clinical parameters: patients were divided into groups depending on whether their headache was sensitive to their menstruation cycle (30.87 ± 7.21, *n* = 16) or not (36.84 ± 8.84, *n* = 20; *p* < 0.014); and the subjective intensity of their headache, such as moderate (28.84 ± 6.63, *n* = 7, VAS 5–7) or severe (35.72 ± 8.63, *n* = 29, VAS 8–10; *p* < 0.017). Mild linear relationships were realised between the attack frequency and concentration changes of XA (*p* < 0.047, R = − 0.379) and MELA (*p* < 0.024, R = − 0.427). Significant correlations were found between the age of healthy controls and their plasma XA (p < 0.024 R = − 0.386) and MELA (*p* < 0.035, R = − 0.387) levels, but this was not observable in migraineurs (interictal XA and age: *p* < 0.077, R = − 0.339) (Fig. 4). The other examined clinical features did not show correlation with the plasma concentrations of metabolites.

### Associations between altered plasma Trp metabolites in the ictal periods of all migraine patients and their clinical parameters

**Fig. 5:**
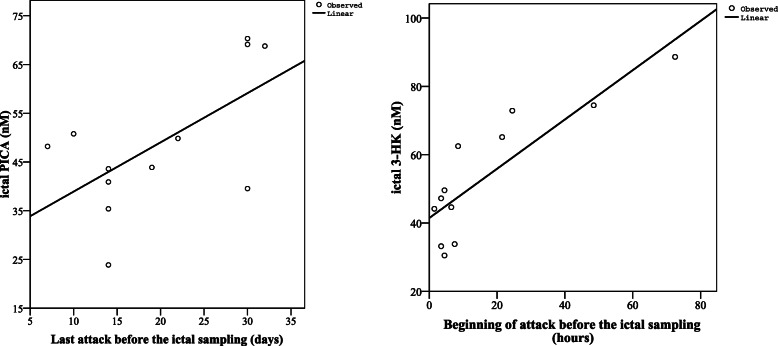
Associations between altered concentrations of PICA and 3-HK and ictal characteristics of all migraine patients (n = 12). PICA: Picolinic acid, 3-HK: 3-hydroxykynurenine

We found positive correlation between the ictal concentrations of PICA and the last attack before the ictal sampling (*p* < 0.031, R = 0.620), as well as between the ictal concentrations of 3-HK and the beginning of attack before ictal sampling (*p* < 0.0004, R = 0.853). However, only 12 samples could be included in this analysis, which highly limits the results and conclusions (Fig. 5).

## Discussion

In this study, we present a detailed analysis of Trp metabolism including kynurenine, 5-HT and MELA pathways in the peripheral plasma of episodic migraineurs, with special attention to alterations in the interictal/ictal periods and clinical associations. The importance of Trp, 5-HT and MELA has already been identified in migraine [8, 9, 11], however the KP has received little attention in human studies. The KP is the main branch (95%) of Trp catabolism and its several metabolites can influence different pain-related mechanisms, including glutamate-mediated neurotransmission, immunological or antioxidant processes. Simultaneous investigation of the different routes of Trp metabolism can get us closer to the metabolomic alterations characteristic of migraine.

Our results show that the entire metabolic route is significantly depressed during the interictal period in migraineurs, but a tendency of elevated metabolite levels was found during the ictal period. Our hypothesis is that decreased Trp metabolism can enhance the susceptibility of patients to the formation of headache attacks. Since all metabolites are closely related to each other, focusing on the provoking effect of individual metabolites might be an oversimplification. More likely, altered metabolite levels paired with other known/unknown trigger mechanisms might have an additive effect that can culminate in an attack when the threshold has been exceeded [37].

The headache attack might be a response to cerebral energy deficit or oxidative stress, which can manifest in impairment in different structures of the brain or striated muscles, elevated lactate levels of the occipital cortex in patients with aura, mitochondrial enzyme dysfunctions and abnormalities in glucose/lipid metabolism [38]. These alterations possibly derive from several peripheral disturbances, which might pose initiating factors for neurological disorders.

For instance, if the Trp and its metabolites have lower concentration in the CNS of migraineurs, the brain is supposedly able to take up Trp from the periphery through large neutral amino acid transporters [39, 40] in order to synthesise the required molecules. This mechanism can protect the brain from damage (also known as counterbalancing excitotoxicity), maintain the central homeostasis, but generate a totally imbalanced Trp pathway manifesting in low peripheral metabolite levels. The extremely reduced metabolite concentrations (as energy deficit) can easily lead to elevated intracellular Ca^2+^ levels through several complex mechanisms, which can finally initiate an attack through enhanced hypersensitization in the CNS [41].

**Fig. 6:**
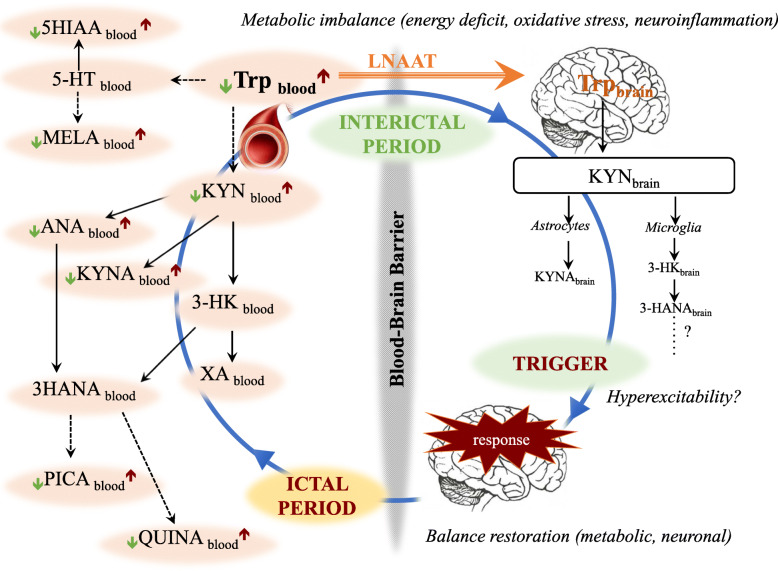
Scheme of altered Trp catabolism and potential mechanisms during interictal/ictal periods in episodic migraineurs. LNAAT: large neutral amino acid transporter, Trp: Tryptophan, 5-HT: 5-hydroxytryptamine, 5-HIAA: 5-hydroxy-indoleaceticacid, MELA: Melatonin, KYN: L-kynurenine, KYNA: Kynurenic acid, ANA: Anthranilic acid, 3-HK: 3-hydroxykynurenine, XA: Xanthurenic acid, 3-HANA: 3-hydroxy-anthranilic acid, PICA: Picolinic acid, QUINA: Quinolinic acid. Continuous arrow: direct step in the pathway, Dashed arrow: indirect step in the pathway

The centrally accumulated metabolites can release into the blood in order to redistribute the molecules in the periphery. This might be a comprehensive regulating feedback mechanism, the aim of which is to reconstitute the control level protecting the organism without the excessive exhaustion (Fig. 6). However, there is a complex relationship between peripheral and central Trp pathways, so it is unknown whether plasma concentrations assessed in our study reflect brain concentrations of metabolites. While changes in the plasma can occur within a larger range influenced by certain drugs or diet, metabolite levels in the brain are more constant. It is presumed that decreased levels of metabolites in the peripheral plasma are attributable to 1) less Trp intake or 2) that the brain can reserve the needed metabolites for itself from the blood. The latter depends on the 1) permeability of blood-brain barrier (BBB), the integrity of which is subject to debate in migraine [42] and 2) the ability of certain molecules to traverse the BBB; in this case the Trp, KYN and 3-HK are considered as well penetrating, actively transported metabolites, while other knyurenine agents can pass via passive diffusion [39].

The function of these metabolites is complicated and hard to pin down due to their widespread involvement in different mechanisms. Most of the investigated molecules are considered protective factors (Trp, KYN, KYNA, ANA, PICA, MELA) and their elevated levels in the periphery might be a consequence of the migraine attack, possibly aimed to restore the balance of energy homeostasis and protect the organism against harmful effects. However, there are molecules with more ambiguous roles (5-HIAA or 3-HK, 3-HANA and QUINA), the increased expression of which might contribute to the formation or aggravation of headache attacks.

Both migraine and the KP were extensively investigated in association with exercise and dietary habits, subsequently energy and metabolic homeostasis and immune system [38, 43, 44]. Although their relationship is not clear as of today, long-lasting peripheral changes and imbalance in Trp metabolism can putatively participate in the development of migraine.

### Role of Trp, 5-HT, 5-HIAA and MELA in migraine

In humans, dietary Trp is utilised either for the synthesis of proteins, 5-HT and MELA or oxidised via the KP at the periphery. In this study, the individual dietary habits of our participants were not assessed in detail, but none of them followed extreme diet regimes. The cause of decreased Trp level during attack-free periods is unknown, but evidence exists for associations between depleted Trp condition/less Trp intake with more frequent headache, glare/light-induced pain and vegetative symptoms [10]. Moreover, approximately 1 g per day Trp consumption seems to reduce the risk of migraine by half [11], therefore exogenous Trp intake should be considered in following studies. Consequently, the decreased Trp concentration can result in the diminished synthesis of consecutive metabolites: e.g. 5-HT, the reduction of which can aggravate phonophobia [10].

Exact correlation has not been found between Trp hydroxylase gene polymorphisms and migraine in genetic studies yet [45], but several studies provide evidence that fluctuating levels of 5-HT both in the periphery and brain take part in the initiation of headache attacks [6, 8, 46–48]. Our results concerning the higher interictal plasma 5-HT level may also explain the decreased Trp level in the attack-free phase and it can serve as protective factor, if we agree with theories that 1) the 5-HT level in platelets can suddenly fall down during attack [49] and 2) the intravenously administered 5-HT is able to abolish both the spontaneous and induced migraine attacks [50]. However, these 5-HT data were not so homogenous and significant alterations were not detected between groups, therefore detailed and exact conclusions could not be drawn.

However, 5-HIAA seems to be an active metabolite, because 1) its decreased level in the CSF has previously been demonstrated in patients with depressive disorders and idiopathic pain syndromes [51] and it is also used as indicator of serotonergic activity [52]*,* 2) its exogenous administration resulted in potentiated thermal hyperalgesia and 3) depressed 5-HIAA content was measured in nerve tissues, if 5-HT synthesis inhibitor was applied as pretreatment in a Complete Freund’s Adjuvant (CFA)-induced peripheral inflammation model [53]. The interictally decreased, but ictally increased 5-HIAA plasma levels observed in our study are not fully in line with previously detected alterations in migraineurs [46], which shows that the exact role of 5-HIAA has not been clarified yet. Higher 5-HIAA levels seem to be a triggering or aggravating factor of headache, which may originate from interictally increased 5-HT levels (paired with an unknown trigger the metabolism of 5-HT can shift toward the 5-HIAA resulted in the formation of attack, while the concentration of plasma 5-HT decrease).

5-HT is converted indirectly to MELA, which is a potent antioxidant and takes part in the regulation of circadian rhythm and depression. In migraineurs, decreased MELA concentrations were found in nocturnal plasma samples [54], which showed an association with hormonal changes [7]. The importance of MELA in migraine was realised when patients exhibited a normalized biological clock, diminished headache frequency and reduced headache intensity following MELA administration. It is presumed that the migraine attack might be a response to the irregularity of pineal cycles [9, 55].

This concept fits our results, wherein the interictally decreased MELA concentration might be a trigger of subsequent attacks through the absence of its protective effect. The accumulated triggering events can evoke headache attacks, which are accompanied by increasing MELA concentration in the periphery in order to reach the normal level and eliminate pain through a positive feedback mechanism. However, more frequent recurrent attacks come with lower interictal MELA levels (mild negative association) according to our results, which might be a sign of impaired re-balancing of MELA levels in migraine. Additionally, age-related lower MELA concentrations were found in our controls suggesting that circadian disturbances are more frequent in the older population due to increased susceptibility for impaired redox homeostasis, suppressed MELA levels and subsequently chronodisruption [56]. Presumably, this association can stay in hidden in migraineurs due to their other specific alterations.

### Role of the KP in migraine

The relevance of kynurenine metabolites in migraine is only partially elucidated but the antiglutamatergic [4], anti-inflammatory [57] antioxidant [58] and antinociceptive features of KYNA are established. It acts on the peripheral and central arms of the TS, therefore KYNA or its derivatives can diminish nociception and hyperalgesia [59, 60], block the cellular activity of migraine generators [61] and inhibit sensitization and neuro-inflammation [29, 62] in different preclinical models. Although the peripheral modulating action of KYNA on sensory and trigeminal nerves is very important in alleviating the mechanisms of migraine [63], its central effect is not so direct because of its poor ability to cross the BBB. KYN penetrates the brain more efficiently, which can fuel the KP giving a basis for conversion of downstream metabolites (e.g. KYNA) in the CNS [27, 28].

There is evidence that endogenous KYNA synthesis in the dura may contribute to the modulation of NMDA receptor function [23]; additionally, lower KYNA concentrations may be accompanied by the overactivation of NMDA receptors [31]. Considering this, decreased KYNA levels observed in the headache free period of our patients can evoke attacks through glutamate excess. Several studies confirm that glutamate is involved in the processes of migraine [1], although there are mixed results concerning the glutamate level during interictal/ictal periods [3, 64]. The ictally elevated glutamate concentration in the CSF may reflect persistent neuronal hyperexcitability, while glutamate receptor antagonists are able to mitigate both the headache [65] and aura phase [66]. The essential role of glutamate in the development of CSD is supported by multiple studies [67, 68], moreover the elevated KYNA levels observed in the ipsilateral hemispheres of rat after triggered CSD [69] call attention to a protective feedback mechanism. This defensive phenomenon might underlie higher interictal KYNA levels identified in migraine patients whose headache was not related to menstruation cycle compared to patients whose migraine was sensitive to hormonal changes. Both migraine attacks and the KP are highly influenced by the female hormone system. In a preclinical study elevated CSD frequency was observed in female rats, when estrogen levels were higher [70], but treatment with KYN significantly decreased the frequency of CSD due to the increased cortical levels of KYNA. Females have a higher intrinsic capacity to synthesize KYNA in baseline conditions than males due to the difference between sex hormones on the synthesizing enzymes involved in Trp metabolism [30]. This suggests that the expression of KYNA is exposed to the impact of hormones in menstruation dependent migraineurs resulting in significant decreased interictal KYNA concentration. If the hormonal effects are not so intense, the level of KYNA might stay in a higher, protective range in the attack free phase. Similarly, in another study, significantly lower MELA levels were detected throughout the ovarian cycle in menstruation-related MWoA patients than in controls [7, 71].

The higher interictal KYNA level in patients with higher headache intensity during the attack may represent a compensatory event aimed at reinforcing endogenous analgesic mechanisms. During severe headache, the organism generates a large amount of protective compounds in order to eliminate the pain. However, further investigation is required to clarify the relationship between KYNA levels and VAS due to certain limitations (few number of patients, subjectivity of VAS). The endogenous compensatory effect of elevated KYNA level was described in an animal experiment, where both glutamate and KYNA levels in the TNC increased significantly during the early state of peripheral inflammation induced by CFA. Glutamate excess can exacerbate pain, while KYNA is able to inhibit glutamate excitotoxicity via NMDA receptor antagonism. Altered glutamate metabolism in migraine is undisputable and KYNA can be protective against trigeminal hyperactivity, but medications targeting the glutamatergic pathway have limited time interval [26].

Beside glutamatergic processes, there are other targets, mechanisms and metabolites of the KP [31, 32], which can influence the development of headache disorders. Oxidative stress and excitotoxicity [38], microstructural lesions [72, 73] and immunological alterations [74] are mainly interrelated and both are regulated by kynurenines.

#### Oxidative stress-excitotoxicity-neurodegeneration

In neurodegenerative disorders, KYN, PICA and ANA serve as protective agents through their ability to block cholinergic toxicity, neuronal loss and glial proliferation. Although both inhibitory and excitatory properties of PICA have been reported [75, 76], it is mainly considered as an endogenous neuroprotective compound within the brain [77, 78] due to its ability to chelate metal ions and effectively inhibit neurotoxicity at the NMDA receptor [79]. In our study the level of PICA showed significant reduction in MWA patients compared to healthy controls. Investigation of MWA and MWoA in separate groups is justified by functional and structural differences between the two subtypes [73, 80], and the interactions between 5-HT pathway and presence of aura/ovarian hormones both in CSD model [70] and in MWA patients [81], but we did not find significant differences. The positive association we found between the ictal level of PICA and the number of days since the last migraine attack points to protective features of PICA and suggests that in patients who recently had a migraine attack, systemic PICA expression is exhausted, bringing about only a slight PICA increase in the plasma during the analysed attack. Presumably, elevated PICA expression occurred during the last attack but fell again suddenly due to triggers contributing to the next attack. However, this interpretation has limitations in this study (number of samples, lack of individual consecutive inter-ictal periods).

Lower KYNA, PICA and/or XA concentrations were measured in patients with depressive episodes [82, 83] and in different stages of schizophrenia [84], which confirm the importance of accurate and detailed differential diagnosis, especially in case of psychiatric involvement. Not only disease-, but also age-specific correlations were detected between the activity of cerebral KP and neurogenesis/excitotoxicity [85], where XA was prominent [86]. Similarly, we found negative correlation between XA levels and age in our control population. The endogenous protective features of XA can explain this association: it has important function in synaptic signalling transports, effective reduction of 3-HK neurotoxicity by transamination [87], moreover it has sedative and analgesic properties in higher doses [88]. Our data showed a weak negative correlation between the interictal concentrations of XA and attack frequency suggesting the absence of protective function: during recurrent attacks, the peripheral concentration of XA cannot return to the control level, causing persistent low levels in the blood.

Generally, 3-HK, 3-HANA and QUINA are categorised as pro-oxidant and proinflammatory molecules, elevated levels of which can generate reactive oxygen species (ROS) and neuronal toxicity, resulting in neuronal death [89] via NMDA agonism, endogenous glutamate release/uptake inhibition [90], enhanced lipid peroxidation [91] and altered BBB integrity [92]. Although these alterations are mainly characteristic for neurodegenerative disorders [93, 94], microstructural alterations observed in the white matter of the migraine brain (“maladaptive plasticity”) can be considered as the prelude of degeneration.

In neurodegenerative disorders, 3-HK and QUINA tend to function as harmful substances [33, 95, 96], but there are controversial results concerning their toxicity, which might explain the depressed Trp metabolism observed in our study. 3-HK and 3-HANA can behave as prooxidant and antioxidant molecules as well [97, 98]. 3-HANA and QUINA have the ability to form coordination complexes with iron or copper [99], thereby they are able to prevent cell damage caused by ROS [100] and maintain the redox homeodynamic equilibrium [101]. 3-HANA has anti-inflammatory and immune/neuroprotective effects [102], while QUINA can attenuate CSD in the rat cortex by means of NMDA desensitization [103]. 3-HK has dual redox modulatory activity depending on its concentration range. Its protective action is related to the stimulation of glutathione- and superoxide systems [104].

In our study, ictal levels of 3-HK showed positive correlation with the elapsed hours since the beginning of attack, which suggests a compensatory mechanism, if the function of 3-HK is considered as more protective than neurotoxic. The level of 3-HK is low during the beginning of the attack, but it begins to elevate afterwards in order to restore metabolic balance. Otherwise, if 3-HK has toxic effects, it can contribute to the prolongation of headache. This is in line with findings of reduced Trp, XA 3-HANA and QUINA levels in connection with neurodegeneration [101], which show that kynurenines have relatively complex milieu-dependent effects. It is not clear how alterations of KP vary between different neurological conditions.

#### Neuro-immunological relationship

The indoleamine 2,3-dioxygenase (IDO), as a rate-limiting enzyme of the KP, has a crucial role in maintaining the neuro-immunological homeostasis [105], which means it is related to the development of migraine [106]. Enhanced activity of IDO and pro-inflammatory changes controlled by several immunological factors were observed in migraineurs [107], possibly reflecting a shift of Trp toward the KP, which results in lower plasma 5-HT availability in the interictal period of patients. This theory was supported by the finding that administration of niacin improved migraine symptoms [108] due to the shift of Trp metabolism toward 5-HT synthesis. Our data may suggest that the interictally higher 5-HT level can drain the Trp resource; thereby the increased 5-HT concentration can maintain the interictal, “resting” state (without symptoms – see as niacin effect). Meanwhile, the plasma Trp resource decreases further because of the transport of Trp to the brain in order to maintain the central homeostasis, resulting in an attenuated Trp catabolism toward the KP. Eventually, the peripheral Trp concentration reaches an extreme low level, when neither the plasma 5-HT nor the brain Trp level is high enough to prevent the headache attack. Apart from Trp, ANA has a protective, immunological effect, exhibiting iron chelating, ROS scavenging and antioxidant features. An ANA derivative compound was able to suppress the activation of monocytes and interferon-gamma induced microglial nitric oxide synthase activity [109]. In our study, significantly decreased concentrations of ANA were observed during the attack free period in MWoA patients (73.11%) compared to healthy controls (100%), which elevated remarkably during the ictal period (108.62%). Low ANA levels might be a contributing factor to elicit the next attack, while its high expression during the ictal period might be a positive feedback response. This elevation is more moderate than what Curto and co-workers observed in the serum of chronic migraineurs [31], but it can prove the importance of ANA.

#### KP and its relation with other systems

Activation of the KP might also be associated with migraine-related peptidergic systems. In our previous study we showed that concentrations of pituitary adenylate cyclase-activating polypeptide 1–38 (PACAP1–38) and calcitonin gene-related peptide (CGRP) decreased in the peripheral plasma during the interictal period in migraineurs, while their levels increased during attacks [110]. An association between the KP and PACAP system was revealed in a rat model of activated TS: pre-treatment with KYNA and its derivative were able to diminish the expression of PACAP1–38 both at proteomic and transcriptomic levels in the peripheral plasma and brain tissues [5]. It is hypothesised that decreasing expression of TRP metabolites can generate an increase in not only glutamate levels but also PACAP1–38 and CGRP concentrations, which can contribute to the formation of migraine attacks. However, this needs further investigations.

## Conclusion

Since migraine is a multifactorial disease, targeting complex pathways could be advantageous to improve therapeutic strategies. It seems that chronically low peripheral levels of TRP metabolites can initiate molecular disturbances underlying migraine pathomechanism, which may participate in triggering events (e.g. glutamate excess, oxidative stress, altered immune functions and neuroinflammation) that can induce hyperexcitability and culminate in headache attacks. The attack itself can be considered as a response to the unbalanced peripheral energy metabolism and the consequent increase in levels of kynurenine, 5-HT and MELA metabolites may serve as protective factor. Our systematic and detailed analysis revealed associations between Trp metabolism and clinical features of migraine, which may lead to new therapeutic options in the future.

## Supplementary Information

**Additional file 1: Supplementary Description 1**. Details of the PLS-LDA method.

## Data Availability

Data concerning metabolite levels and clinical characteristics are available in the Supplementary file.

## Abbreviations

3-HANA: 3-hydroxyanthranilic acid
3-HK: 3-hydroxykynurenine
5-HIAA: 5-hydroxyindoleacetic acid
ANA: Anthranilic acid
CSD: Cortical spreading depression
FA: Formic acid
IQR: Interquartile range
KP: Kynurenine pathway
KYN: l-kynurenine
KYNA: Kynurenic acid
LOD: Limit of detection
LOQ: Limit of quantification
MELA: Melatonin
MWA: Migraine with aura
MWoA: Migraine without aura
NMDA: N-methyl-D-aspartate
PLS-LDA: Partial least squares–linear discriminant analysis
PFP: Pentafluorophenyl
PICA: Picolinic acid
QUINA: Quinolinic acid
TNC: Nucleus trigeminus caudalis
Trp: tryptophan
TS: Trigeminovascular system
UHPLC–MS/MS: ultra high-performance liquid chromatography–tandem mass spectrometry
VAS: Visual analogue scale
XA: Xanthurenic acid
