# Supplementary material for: Clinical relevance of depressed kynurenine pathway in episodic migraine patients: potential prognostic markers in the peripheral plasma during the interictal period
Source: J Headache Pain. 2021 Jun 25;22(1):60. doi: 10.1186/s10194-021-01239-1 (PMC8229298; doi:10.1186/s10194-021-01239-1)
Supplement: Supplementary file 1 — Additional file 1: Supplementary Description 1. Details of the PLS-LDA method. [file 10194_2021_1239_MOESM1_ESM.docx]

**Supplementary file**

**Supplementary Description 1.**

PLS aims to find a decomposition of a set of dependent (*Y*) and independent (*X*) variables such that the covariance of the factors of *X* and *Y* are maximal. The decomposition results in orthogonal components or latent variables (weighted linear combinations of the independent variables in *X*) that can be used to predict *Y*. These latent variables serve as input to the LDA, which uses them to further reduce the dimensionality of the data while achieving maximum class separability, thus classifying metabolite profiles as belonging to migraine patients or healthy participants in our case. Variable selection can then be performed to identify prominent metabolites in the model; for this, we use the variable importance in projection (VIP) score, which is the sum of squared PLS weights weighted by the variance the latent variables explain in the data. Important metabolites were selected on the basis of having a VIP score of >1 according to previous descriptions of the method. We chose the model with the least root mean square error of the model fit.

**Supplementary Table 1. LODs, LOQs and the parameters of the calibration curves for twelve analytes in “blank” human plasma.**

| Analytes | LOD | LOQ | Regression equation | Regression correlation | Linearity range |
| --- | --- | --- | --- | --- | --- |
|  | nM | |  |  | nM |
| 5-HT | 9.754 | 29.557 | *Y* = 0.0006*x* − 0.0053 | 0.9998 | 31.25–1000 |
| KYN | 1.206 | 3.654 | *Y* = 0.3700*x* − 0.0369 | 0.9997 | 312.5–10000 |
| 3-HANA | 0.730 | 2.213 | *Y* = 0.0081*x* + 0.0009 | 0.9999 | 7.8125–250 |
| Trp | 1.920 | 5.818 | *Y* = 0.4418*x* − 0.0311 | 0.9996 | 6250–200000 |
| 5-HIAA | 0.506 | 1.533 | *Y* = 0.0113*x* − 0.0019 | 0.9998 | 7.8125–250 |
| ANA | 0.753 | 2.282 | *Y* = 0.0013*x* + 0.0029 | 0.9998 | 4.6875–150 |
| KYNA | 0.089 | 0.271 | *Y* = 0.0223*x* + 0.0042 | 0.9998 | 4.6875–150 |
| XA | 0.136 | 0.413 | *Y* = 0.0391*x* + 0.0071 | 0.9998 | 3.125–100 |
| 3-HK | 0.580 | 1.759 | *Y* = 0.0126*x* − 0.0505 | 0.9995 | 9.375–300 |
| MELA | 0.103 | 0.312 | *Y* = 0.2727*x* − 0.0003 | 0.9995 | 0.078125–2.5 |
| PICA | 0.909 | 2.755 | *Y* = 0.0116*x* + 0.0665 | 0.9999 | 4.6875–150 |
| QUINA | 5.608 | 16.994 | *Y* = 0.0019*x* + 0.2039 | 0.9996 | 31.25–1000 |

*LOD: limit of detection, LOQ: limit of quantification, 5-HT: 5-hydroxytryptamine, KYN: l-kynurenine, 3-HANA: 3-hydroxy-anthranilic acid, Trp: Tryptophan, 5-HIAA: 5-hydroxyindoleacetic acid, ANA: Anthranilic acid, KYNA: Kynurenic acid, XA: Xanthurenic acid, 3-HK: 3-hydroxykynurenine, MELA: Melatonin, PICA: Picolinic acid, QUINA: Quinolinic acid*

**Supplementary Table 2. Intra-day precision and accuracy of analysed metabolites with the measured concentrations in nM. The table shows the spiked concentrations and measured concentrations at low, medium and high levels in “blank” human plasma.**

| Quality  Control | Day | Concentration (nM) | Measured concentration (nM) | | |  | Concentration (nM) | Measured concentration (nM) | | |
| --- | --- | --- | --- | --- | --- | --- | --- | --- | --- | --- |
|  |  | 5-HT | Mean ± SD | RSD% | % accuracy |  | KYN | Mean ± SD | RSD% | % accuracy |
| LQC | 1 | 93.75 | 103.4 ± 1.67 | 1.6 | 110.2 |  | 937.5 | 1070.7 ± 36.83 | 3.4 | 114.2 |
|  | 2 |  | 100.6 ± 2.13 | 2.1 | 107.3 |  |  | 1070.1 ± 19.90 | 1.9 | 114.1 |
|  | 3 |  | 103.4 ± 2.77 | 2.7 | 110.2 |  |  | 976.4 ± 46.79 | 4.8 | 104.1 |
| MQC | 1 | 250.0 | 268.9 ± 11.68 | 4.3 | 107.5 |  | 2500.0 | 2780.5 ± 98.66 | 3.5 | 111.2 |
|  | 2 |  | 272.6 ± 6.89 | 2.5 | 109.0 |  |  | 2825.9 ± 59.84 | 2.1 | 113.0 |
|  | 3 |  | 243.8 ± 7.62 | 3.1 | 97.5 |  |  | 2321.7 ± 93.05 | 4.0 | 92.9 |
| HQC | 1 | 750.0 | 758.1 ± 18.01 | 2.4 | 101.1 |  | 7500.0 | 7117.2 ± 253.7 | 3.6 | 94.9 |
|  | 2 |  | 753.7 ± 25.50 | 3.4 | 100.5 |  |  | 7637.3 ± 345.9 | 4.5 | 101.8 |
|  | 3 |  | 758.6 ± 36.80 | 4.9 | 101.1 |  |  | 7320.2 ± 692.7 | 9.5 | 97.6 |
|  |  | 3-HANA |  | | |  | Trp |  | | |
| LQC | 1 | 23.4375 | 24.99 ± 1.41 | 5.6 | 106.6 |  | 18750 | 19143.1 ± 373.5 | 2.0 | 102.1 |
|  | 2 |  | 26.50 ± 1.96 | 7.4 | 113.0 |  |  | 19287.2 ± 811.1 | 4.2 | 102.9 |
|  | 3 |  | 26.46 ± 1.46 | 5.5 | 112.9 |  |  | 19920.2 ± 928.8 | 4.7 | 106.2 |
| MQC | 1 | 62.5 | 69.03 ± 2.63 | 3.8 | 110.4 |  | 50000 | 47515.7 ± 763.6 | 1.6 | 95.0 |
|  | 2 |  | 69.94 ± 4.92 | 7.0 | 111.9 |  |  | 50992.6 ± 778.5 | 1.5 | 102.0 |
|  | 3 |  | 64.51 ± 2.88 | 4.4 | 103.2 |  |  | 49581.9 ± 3214.6 | 6.5 | 99.2 |
| HQC | 1 | 187.5 | 185.0 ± 5.86 | 3.2 | 98.6 |  | 150000 | 143064.0 ± 5146.8 | 3.6 | 95.4 |
|  | 2 |  | 181.1 ± 4.8 | 2.6 | 96.5 |  |  | 144439.4 ± 4733.3 | 3.3 | 96.3 |
|  | 3 |  | 174.0 ± 4.61 | 2.6 | 92.7 |  |  | 155373.3 ± 4089.1 | 2.6 | 103.6 |
|  |  | 5-HIAA |  |  |  |  | ANA |  |  |  |
| LQC | 1 | 23.4375 | 20.01 ± 0.36 | 1.8 | 85.4 |  | 14.0625 | 15.95 ± 0.26 | 1.6 | 113.4 |
|  | 2 |  | 19.95 ± 0.52 | 2.6 | 85.1 |  |  | 16.12 ± 1.30 | 8.0 | 114.6 |
|  | 3 |  | 21.73 ± 0.61 | 2.8 | 92.7 |  |  | 15.11 ± 2.05 | 13.6 | 107.4 |
| MQC | 1 | 62.5 | 58.12 ± 1.46 | 2.5 | 93.0 |  | 37.5 | 38.53 ± 3.55 | 9.2 | 102.7 |
|  | 2 |  | 58.91 ± 2.06 | 3.5 | 94.3 |  |  | 39.96 ± 1.47 | 3.7 | 106.5 |
|  | 3 |  | 59.75 ± 3.01 | 5.0 | 95.6 |  |  | 37.32 ± 2.95 | 7.9 | 99.5 |
| HQC | 1 | 187.5 | 160.5 ± 4.39 | 2.7 | 85.6 |  | 112.5 | 117.3 ± 9.10 | 7.8 | 104.2 |
|  | 2 |  | 163.4 ± 2.69 | 1.6 | 87.1 |  |  | 117.3 ± 10.70 | 9.1 | 104.2 |
|  | 3 |  | 168.3 ± 3.45 | 2.0 | 89.7 |  |  | 112.7 ± 11.20 | 9.9 | 100.1 |

| Quality  Control | Day | Concentration (nM) | Measured concentration (nM) | | |  | Concentration (nM) | Measured concentration (nM) | | |
| --- | --- | --- | --- | --- | --- | --- | --- | --- | --- | --- |
|  |  | KYNA | Mean ± SD | RSD% | % accuracy |  | XA | Mean ± SD | RSD% | % accuracy |
| LQC | 1 | 14.0625 | 13.61 ± 0.35 | 2.6 | 96.7 |  | 9.375 | 9.19 ± 0.05 | 0.5 | 97.9 |
|  | 2 |  | 12.57 ± 0.21 | 1.7 | 89.4 |  |  | 9.00 ± 0.21 | 2.3 | 96.0 |
|  | 3 |  | 15.01 ± 0.35 | 2.3 | 106.7 |  |  | 8.98 ± 0.53 | 5.8 | 95.7 |
| MQC | 1 | 37.5 | 39.64 ± 1.07 | 2.7 | 105.7 |  | 25.0 | 25.38 ± 0.44 | 1.7 | 101.5 |
|  | 2 |  | 36.71 ± 0.78 | 2.1 | 97.9 |  |  | 24.06 ± 0.68 | 2.8 | 96.2 |
|  | 3 |  | 43.69 ± 0.75 | 1.7 | 116.5 |  |  | 24.37 ± 0.24 | 1.0 | 97.5 |
| HQC | 1 | 112.5 | 110.1 ± 1.55 | 1.4 | 97.8 |  | 75.0 | 71.09 ± 2.52 | 3.5 | 94.8 |
|  | 2 |  | 103.7 ± 0.71 | 0.7 | 92.1 |  |  | 69.42 ± 1.58 | 2.3 | 92.6 |
|  | 3 |  | 129.1 ± 2.50 | 1.9 | 114.8 |  |  | 71.83 ± 3.39 | 4.7 | 95.8 |
|  |  | 3-HK |  |  |  |  | MELA |  | | |
| LQC | 1 | 28.125 | 26.00 ± 0.91 | 3.5 | 92.4 |  | 0.2344 | 0.24 ± 0.02 | 8.4 | 98.7 |
|  | 2 |  | 26.17 ± 0.47 | 1.8 | 93.0 |  |  | 0.25 ± 0.02 | 5.9 | 103.9 |
|  | 3 |  | 28.55 ± 2.90 | 10.1 | 101.5 |  |  | 0.21 ± 0.02 | 6.9 | 87.7 |
| MQC | 1 | 75.0 | 80.49 ± 3.21 | 4.0 | 107.3 |  | 0.625 | 0.64 ± 0.02 | 3.1 | 101.9 |
|  | 2 |  | 81.63 ± 1.03 | 1.3 | 108.8 |  |  | 0.66 ± 0.02 | 2.7 | 104.5 |
|  | 3 |  | 78.14 ± 4.56 | 5.8 | 104.2 |  |  | 0.62 ± 0.04 | 5.9 | 98.3 |
| HQC | 1 | 225.0 | 245.7 ± 5.16 | 2.1 | 109.2 |  | 1.875 | 1.75 ± 0.04 | 2.0 | 93.2 |
|  | 2 |  | 253.8 ± 7.05 | 2.8 | 112.8 |  |  | 1.78 ± 0.03 | 1.3 | 94.6 |
|  | 3 |  | 228.0 ± 10.13 | 4.4 | 101.3 |  |  | 1.78 ± 0.12 | 6.7 | 94.9 |
|  |  | PICA |  | | |  | QUINA |  | | |
| LQC | 1 | 14.0625 | 13.85 ± 0.57 | 4.1 | 98.5 |  | 93.75 | 95.13 ± 5.77 | 6.1 | 101.5 |
|  | 2 |  | 14.16 ± 0.47 | 3.3 | 100.7 |  |  | 92.90 ± 8.28 | 8.9 | 99.1 |
|  | 3 |  | 13.66 ± 0.78 | 5.7 | 97.1 |  |  | 102.2 ± 1.41 | 1.4 | 108.9 |
| MQC | 1 | 37.5 | 34.10 ± 1.21 | 3.5 | 90.9 |  | 250.0 | 252.3 ± 8.39 | 3.3 | 100.9 |
|  | 2 |  | 32.39 ± 1.04 | 3.2 | 86.4 |  |  | 241.0 ± 11.23 | 4.7 | 96.4 |
|  | 3 |  | 33.94 ± 2.13 | 6.3 | 90.5 |  |  | 230.9 ± 3.40 | 1.5 | 92.3 |
| HQC | 1 | 112.5 | 108.9 ± 2.57 | 2.4 | 96.8 |  | 750.0 | 781.1 ± 2.20 | 0.3 | 104.1 |
|  | 2 |  | 112.7 ± 4.21 | 3.7 | 100.1 |  |  | 776.7 ± 13.99 | 1.8 | 103.6 |
|  | 3 |  | 101.0 ± 3.74 | 3.7 | 89.7 |  |  | 806.1 ± 5.15 | 0.6 | 107.5 |

*Data is shown as mean ± SD.*

*SD: standard deviation, RSD: relative standard deviation, LQC: low-level quality control, MQC: medium-level quality control, HQC: high-level quality control, 5-HT: 5-hydroxytryptamine, KYN: l-kynurenine, 3-HANA: 3-hydroxy-anthranilic acid, Trp: Tryptophan, 5-HIAA: 5-hydroxyindoleacetic acid, ANA: Anthranilic acid, KYNA: Kynurenic acid, XA: Xanthurenic acid, 3-HK: 3-hydroxykynurenine, MELA: Melatonin, PICA: Picolinic acid, QUINA: Quinolinic acid*

**Supplementary Table 3. Inter-day precision and accuracy for Trp and eleven metabolites with the measured concentrations in nM at low, medium and high levels in “blank” human plasma.**

| Quality Control | Concentration (nM) | Measured concentration (nM) | | |  | Concentration (nM) | Measured concentration (nM) | | |
| --- | --- | --- | --- | --- | --- | --- | --- | --- | --- |
|  | 5-HT | Mean ± SD | RSD% | % accuracy |  | KYN | Mean ± SD | RSD% | % accuracy |
| LQC | 93.75 | 102.4 ± 2.44 | 2.4 | 109.2 |  | 937.5 | 1039.1 ± 56.73 | 5.5 | 110.8 |
| MQC | 375.0 | 261.8 ± 15.62 | 6.0 | 104.7 |  | 2500.0 | 2642.8 ± 250.2 | 9.5 | 105.7 |
| HQC | 750.0 | 756.8 ± 25.30 | 3.3 | 100.9 |  | 7500.0 | 7358.2 ± 480.7 | 6.5 | 98.1 |
|  | 3-HANA |  | | |  | Trp |  | | |
| LQC | 23.4375 | 25.98 ± 1.65 | 6.3 | 110.8 |  | 18750 | 19450.2 ± 759.6 | 3.9 | 103.7 |
| MQC | 62.5 | 67.83 ± 4.11 | 6.1 | 108.5 |  | 50000 | 49363.4 ± 2316.6 | 4.7 | 98.7 |
| HQC | 187.5 | 180.0 ± 6.64 | 3.7 | 96.0 |  | 150000 | 147625.6 ± 7140.1 | 4.8 | 98.4 |
|  | 5-HIAA |  | | |  | ANA |  | | |
| LQC | 23.4375 | 20.56 ± 0.98 | 4.7 | 87.7 |  | 14.0625 | 15.73 ± 1.36 | 8.6 | 111.8 |
| MQC | 62.5 | 58.93 ± 2.17 | 3.7 | 94.3 |  | 37.5 | 38.60 ± 2.77 | 7.2 | 102.9 |
| HQC | 187.5 | 164.1 ± 4.67 | 2.8 | 87.5 |  | 112.5 | 115.7 ± 9.65 | 8.3 | 102.8 |
|  | KYNA |  | | |  | XA |  | | |
| LQC | 14.0625 | 13.73 ± 1.09 | 7.9 | 97.6 |  | 9.375 | 9.05 ± 0.32 | 3.4 | 96.5 |
| MQC | 37.5 | 40.01 ± 3.10 | 7.7 | 106.7 |  | 25.0 | 24.60 ± 0.74 | 3.0 | 98.4 |
| HQC | 112.5 | 114.3 ± 11.41 | 10.0 | 101.6 |  | 75.0 | 70.78 ± 2.58 | 3.6 | 94.4 |
|  | 3-HK |  | | |  | MELA |  | | |
| LQC | 28.125 | 26.90 ± 2.01 | 7.5 | 95.6 |  | 0.2344 | 0.23 ± 0.03 | 9.8 | 96.8 |
| MQC | 75.0 | 80.09 ± 3.33 | 4.2 | 106.8 |  | 0.625 | 0.64 ± 0.03 | 4.5 | 101.6 |
| HQC | 225.0 | 242.5 ± 13.26 | 5.5 | 107.7 |  | 1.875 | 1.77 ± 0.07 | 3.8 | 94.2 |
|  | PICA |  | | |  | QUINA |  | | |
| LQC | 14.0625 | 13.89 ± 0.60 | 4.3 | 98.8 |  | 93.75 | 96.72 ± 6.72 | 6.9 | 103.2 |
| MQC | 37.5 | 33.48 ± 1.61 | 4.8 | 89.3 |  | 250.0 | 241.4 ± 11.84 | 4.9 | 96.5 |
| HQC | 112.5 | 107.5 ± 6.05 | 5.6 | 95.5 |  | 750.0 | 787.9 ± 15.64 | 2.0 | 105.1 |

*Data is shown as mean ± SD.*

*SD: standard deviation, RSD: relative standard deviation, LQC: low-level quality control, MQC: medium-level quality control, HQC: high-level quality control, 5-HT: 5-hydroxytryptamine, KYN: l-kynurenine, 3-HANA: 3-hydroxy-anthranilic acid, Trp: Tryptophan, 5-HIAA: 5-hydroxyindoleacetic acid, ANA: Anthranilic acid, KYNA: Kynurenic acid, XA: Xanthurenic acid, 3-HK: 3-hydroxykynurenine, MELA: Melatonin, PICA: Picolinic acid, QUINA: Quinolinic acid.*

**Supplementary Table 4. The recoveries in “blank” human plasma, expressed as mean % ± RSD.**

| Analytes | % recovery ± RSD |
| --- | --- |
| 5-HT | 96.41 ± 4.03 |
| KYN | 95.15 ± 8.59 |
| 3-HANA | 99.39 ± 3.55 |
| Trp | 91.92 ± 6.02 |
| 5-HIAA | 93.73 ± 4.55 |
| ANA | 101.14 ± 8.53 |
| KYNA | 93.48 ± 4.74 |
| XA | 95.80 ± 7.17 |
| 3-HK | 90.28 ± 8.17 |
| MELA | 90.54 ± 7.12 |
| PICA | 92.79 ± 5.09 |
| QUINA | 101.81 ± 2.25 |

*RSD: relative standard deviation, 5-HT: 5-hydroxytryptamine, KYN: l-kynurenine, 3-HANA: 3-hydroxy-anthranilic acid, Trp: Tryptophan, 5-HIAA: 5-hydroxyindoleacetic acid, ANA: Anthranilic acid, KYNA: Kynurenic acid, XA: Xanthurenic acid, 3-HK: 3-hydroxykynurenine, MELA: Melatonin, PICA: Picolinic acid, QUINA: Quinolinic acid.*

**Supplementary Table 5. Differences in plasma levels of Trp metabolites between healthy controls and migraine patients with aura/without aura.**

| **Metab. (nM)** | **Groups: Median ± IQR** | | | **Significant p-value between groups** |
| --- | --- | --- | --- | --- |
|  | **0. Healthy Control** | **1. Interictal Migraine With Aura** | **2. Interictal Migraine Without Aura** |  |
| **Trp** | 53203.65 **±** 14599.34 | 47835.39 **±** 8132.08 | 44009.74 **±** 13499.17 | 0–2: **0.018** |
| **KYN** | 2505.77 **±** 902.53 | 2268.30 **±** 616.41 | 2043.32 **±** 705.02 | 0–2: **0.004** |
| **KYNA** | 39.59 **±** 15.32 | 41.57 **±** 19.84 | 33.74 **±** 14.05 | 0–2: **0.019** |
| **ANA** | 47.12 **±** 27.79 | 43.00 **±** 30.19 | 36.31 **±** 17.35 | 0–2: **0.006** |
| **3-HK** | 47.29 **±** 27.89 | 51.39 **±** 12.94 | 42.68 **±** 19.00 | – |
| **XA** | 17.42 **±** 13.46 | 11.32 **±** 5.47 | 12.84 **±** 9.30 | 0–1: **0.032** |
| **3-HANA** | 47.78 **±** 24.59 | 42.80 **±** 14.12 | 44.11 **±** 23.07 | – |
| **PICA** | 46.61 **±** 25.63 | 33.72 **±** 17.84 | 35.97 **±** 13.32 | 0–1: **0.038**  0–2: **0.025** |
| **QUINA** | 252.92 **±** 169.69 | 213.54 **±** 193.01 | 196.01 **±** 94.55 | – |
| **5-HT** | 468.11 **±** 710.24 | 409.32 **±** 557.21 | 809.32 **±**560.88 | – |
| **5-HIAA** | 45.31 **±** 20.46 | 38.00 **±** 14.07 | 37.46 **±** 11.30 | – |
| **MELA** | 0.19 **±** 0.13 | 0.17 **±** 0.09 | 0.16 **±** 0.07 | 0–2: **0.018** |

*All significant p-values were added in the table between healthy controls and interictal groups of migraine patients.*

*IQR: interquartile range, Metab.: metabolites, Trp: Tryptophan, KYN: L-kynurenine, KYNA: Kynurenic acid, ANA: Anthranilic acid, 3HK: 3-hydroxykynurenine, XA: Xanthurenic acid, 3HANA: 3-hydroxy-anthranilic acid, PICA: Picolinic acid, QUINA: Quinolinic acid, 5HIAA: 5-hydroxy-indoleaceticacid, MELA: Melatonin*

|  | **Groups: Median ± IQR** | | | **p-value between groups** |
| --- | --- | --- | --- | --- |
|  |  | **Migraine Without Aura** | |  |
| **Metab. (nM)** | **0. Healthy Control** | **1. Interictal period** | **2. Ictal period** |  |
| **Trp** | **53203.65 ±** 14599.34 = 100% | 42627.16 **±** 13780.99 = 80.12% | 45687.30 **±** 12135.15 = 85.87% | 0–1: **0.025** |
| **KYN** | **2505.77 ±** 902.53 = 100% | 1910.39 **±** 613.70 = 76.24% | 2472.79 **±** 808.08 = 98.68% | 0–1: **0.001** |
| **KYNA** | **39.59** **±** 15.32 = 100% | 32.24 **±** 14.56 = 81.44% | 37.33 **±** 12.96 = 94.29% | 0–1: **0.016** |
| **ANA** | **47.12** **±** 27.79 = 100% | 34.45 **±** 15.95 = 73.11% | 51.18 **±** 33.64 = 108.62% | 0–1: **0.007** |
| **3HK** | **47.29** **±** 27.89 = 100% | 40.85 **±** 16.78 = 86.38% | 54.87 **±** 39.61 = 116.03%. | 0–1: 0.322 |
| **XA** | **17.42** **±** 13.46 = 100% | 12.21 **±** 8.30 = 70.09% | 17.61 **±** 17.37 = 101.09% | 0–1: 0.066 |
| **3HANA** | **47.78** **±** 24.59 = 100% | 47.16 **±** 23.03 = 98.70% | 42.40 **±** 19.14 = 88.74% | 0–1: 0.818 |
| **PICA** | **46.61** **±** 25.63 = 100% | 35.81 **±** 14.10 = 76.83% | 46.04 **±** 16.85 = 98.78% | 0–1: **0.030** |
| **QUINA** | **252.92** **±** 169.69 = 100% | 186.53 **±** 97.75 = 73.75% | 239.66 **±** 204.61 = 94.76% | 0–1: **0.035** |
| **5-HT** | **468.11** **±** 710.24  = 100% | 800.27 **±** 534.58  = 170.96% | 491.23 **±** 509.87  = 104.94% | 0–1: 0.122 |
| **5HIAA** | **45.31** **±** 20.46 = 100% | 36.14 **±** 9.26 = 79.76% | 41.95 **±** 8.83 = 92.58% | 0–1: **0.025** |
| **MELA** | **0.19** **±** 0.11 = 100% | 0.15 **±** 0.08 = 78.95% | 0.19 **±** 0.08 = 100% | 0–1: **0.023** |

**Supplementary Table 6. Differences in plasma levels of Trp metabolites between healthy controls and interictal/ictal phases of migraine without aura patients.**

*All p-values were added in the table between control and interictal group of migraine without aura patients.*

*IQR: interquartile range, Metab.: metabolites, Trp: Tryptophan, KYN: L-kynurenine, KYNA: Kynurenic acid, ANA: Anthranilic acid, 3HK: 3-hydroxykynurenine, XA: Xanthurenic acid, 3HANA: 3-hydroxy-anthranilic acid, PICA: Picolinic acid, QUINA: Quinolinic acid, 5HIAA: 5-hydroxy-indoleaceticacid, MELA: Melatonin*
